# Study on Impermeability of Foamed Concrete Containing Municipal Solid Waste Incineration Powder

**DOI:** 10.3390/ma15155176

**Published:** 2022-07-26

**Authors:** Yun Dong, Yuanshan Ma, Jinbiao Zhu, Jianchun Qiu

**Affiliations:** 1 Huaiyin Institute of Technology, Faculty of Architecture and Civil Engineering, Huai’an 223001, China; dyun@hyit.edu.cn (Y.D.); zhu01470147@163.com (J.Z.); 2 College of Water Conservancy and Hydropower Engineering, Hohai University, Xikang Road No. 1, Nanjing 210098, China; 3 College of Hydraulic Science and Engineering, Yangzhou University, Yangzhou 225009, China; qiujc@yzu.edu.cn

**Keywords:** municipal solid waste incineration (MSWI) powder, permeability resistance, porosity, compressive strength, foamed concrete

## Abstract

In this paper, the effects of dry density, w/c ratio, and municipal solid waste incineration (MSWI) powder on the multi-scale properties and internal pore structure of foamed concrete were studied by using a single-factor controlled experiment. It was found that an increase in the dry density of foamed concrete could effectively reduce the porosity, leading to the improvement of compressive strength and impermeability and to the reduction of water absorption. The compressive strength, water absorption, and impermeability were mainly affected by the porosity when the w/c ratio changed. With the increase in porosity, the water absorption rate increased, and the compressive strength and impermeability decreased. The addition of MSWI powder caused no obvious change in the overall pore size distribution of the foamed concrete, and there was no significant change in the water absorption and impermeability of the structure. However, because the hydration activity of MSWI powder was lower than that of ordinary Portland cement, the compressive strength of foamed concrete decreased with the increase in MSWI powder.

## 1. Introduction

Foamed concrete is a new type of porous building material formed by pouring and curing a mixture of cementitious material and prefabricated foam through physical foaming or chemical foaming [1]. Foamed concrete produces a large amount of foam in the cement paste through the introduction of chemical admixtures, so the density of the mixture is much lower than that of ordinary concrete. Therefore, foamed concrete has many excellent properties, such as good paste workability, less aggregate consumption, good overall performance after hardening, light and flame retardant properties, excellent sound insulation and heat insulation performance, easy construction, strong freezing–thawing resistance, and sulfate resistance [2,3].

As the advantages of foamed concrete gradually appear, many scholars at home and abroad have carried out relevant research on foamed concrete. The study by Nambiar, E. K. et al. [4] showed that stomatal size and spacing would affect strength and density. The decrease in pore size distribution led to an increase in the strength of the foamed concrete. However, the deformation degree of the pores had no obvious influence on the compressive strength. Wee, T.-H. et al. [5] studied the influence of the w/c ratio on the pore structure of foamed concrete (10–70% gas content) and its influence on the mechanical properties of sandless foamed concrete. When the foam content was the same, the above pore structure parameters changed with the variations in w/c ratio and porosity, affecting the performance of the foamed concrete. The effect of porosity on the elastic modulus of foamed concrete was not as significant as that of compressive strength. Chung, S.-Y. et al. [6] prepared several foamed concrete samples with various supplementary cementitious materials (SCMs) for study, and the results showed that the SCM in volcanic ash could improve the strength of foamed concrete materials by increasing the content of calcium silicate hydrate (C−S−H). In addition, their microstructural study also confirmed that the w/c ratio had an effect on the degree of hydration. Kearsley, E. P. [7] found that the main factor affecting the porosity of foamed concrete was the dry density of foamed concrete rather than the type or content of ash added. Foamed concrete absorbed approximately twice as much water as an equivalent cement paste, regardless of the volume of air introduced, ash type, or ash content. The water vapor permeability increased with the increase in porosity and ash content. Nambiar, E. K. K. et al. [8] prepared foamed concrete with different amounts of fly ash and foam, and the results showed that the water absorption of the foamed concrete increased, but the adsorption value was lower than that of the corresponding conventional concrete (without foam). The adsorption process was related to the type of filler, density, pore structure, and permeation mechanism. Hilal, A. et al. [9] believed that the porosity of foamed concrete increased with the decrease in density and that the critical pore size increased with the increase in density, both of which remarkably influenced the permeability. Aravind, N. R. et al. [10] used foamed concrete and rice husk and used fly ash instead of cement to prepare wall panels. Planar bending tests and compressive strength tests were used to test the strength of the panels. Compared with the control sample with no rice husk, the bending strength and breaking modulus decreased by 18% when the rice husk content was 15%. Adhikary, S. K. et al. [11] tested and studied three different types of lightweight insulation materials, including expanded glass aggregate, silica aerogel, and prefabricated plastic bubbles as aggregate and fine fillers. The influence of aerogel particles on the hydration process of foamed concrete was studied by using scanning microscopy, thermal analysis, and XRD analysis. Tiong, H. Y. et al. [12] used eggshell powder (ESP) as a partial substitute for cement to test the initial surface adsorption rate, adsorbability, ultrasonic pulse speed, and other properties of lightweight foam-based concrete (LFC) with a density of 1300 kg/m^3^. The results indicated that the pore structure, w/c ratio, and admixture had an important influence on the evaluation of the basic performance of foamed concrete.

Municipal solid waste incineration (MSWI) powder is a byproduct of waste incineration that reduces both the volume and mass to less than 20% of those of the original waste. With the consideration of environmental protection and energy savings, MSWI powder has been paid more and more attention in the civil engineering field. Mathews, G. et al. [13] tested the physical and chemical properties of reclaimed sand with MSWI powder. It was found that the use of reclaimed sand as a lightweight concrete aggregate met most the ASTM requirements. However, a large number of pores in the microstructure of the cement paste led to cracks in concrete, reducing the bearing capacity of the structure. Minane, J. R. et al. [14] found that MSWI powder could be used in concrete as a substitute for natural sand because MSWI powder had a similar particle size distribution and specific gravity to those of commonly used natural sand and had a higher water absorption rate (WAb = 7.50%) than that of natural sand. Singh, D. et al. [15] studied the effect of cement and fiber incorporation on the compaction and strength of MSWI. It was found that when cement and fiber were added to MSWI powder, the optimal moisture content of the MSWI powder increased and the maximum dry density of the MSWI powder decreased. Cement with fiber can be used as a modified reinforcement material for MSWI powder. Vegas, I. et al. [16] studied and analyzed the technical applicability of secondary products from three types of waste materials (construction and demolition waste (CDW), Waelz slag, and MSWI powder) in accordance with regulations and standards for use in road construction. The results showed that Waelz slag could be used as a granular structure layer, while CDW was more suitable as a granular material in the roadbed. Saikia, N. et al. [17,18] characterized metallurgical slag from lead production, MSWI powder (0.1–2 mm) from a grate furnace (SF), and slag and fly ash from a fluidized bed incinerator (BFA). It was found that the incorporation of SLG had no adverse effects on the compressive strength of the mortar, while some components of BFA and SF reacted with alkali to form ettringite, aluminum hydroxide, hydrogen, and other expansion products, reducing the compressive strength of the mortar.

According to the above discussion, there have been many studies on MSWI powder at home and abroad, but there are still limitations in the activation of MSWI powder, especially in the preparation of foamed concrete with MSWI powder instead of cement. Therefore, MSWI was ground into powder and added into foamed concrete to study its influence on compressive strength, water absorption, impermeability, and pore structure. Combined with the macro performance test indicators and pore structure parameters, the mechanism of MSWI powder in the internal structure of foamed concrete is discussed through experimental research and mathematical analysis.

## 2. Materials and Test Methods

### 2.1. Material

**Figure 1:**
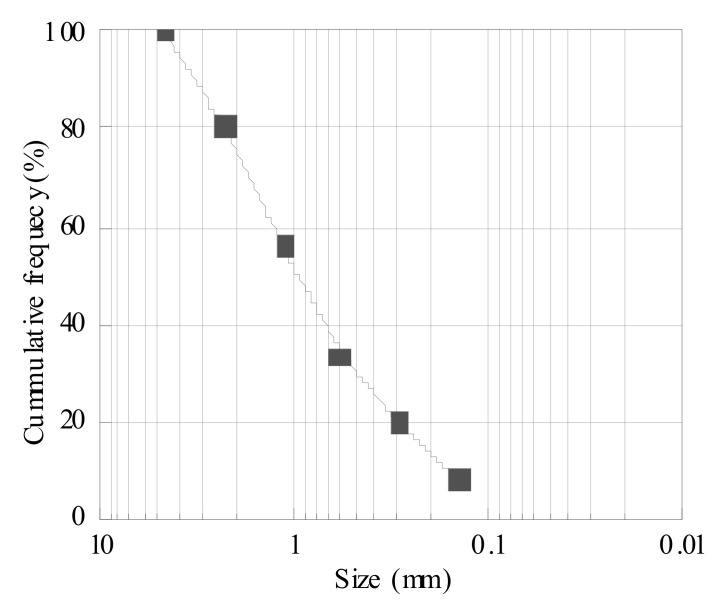
MSWI particle size distribution.

The MSWI powder used in this study was obtained from the Xuyi Household Waste Incineration Power Plant of the Jiangsu Shengyuan Environmental Protection Electric Power Company (Xuyu, China). The basic physical properties and chemical composition are shown in Figure 1 and Table 1. After washing, drying, crushing, and grinding the MSWI powder, a sieve was used for screening, and a micropowder was collected in the chassis of a 200 mesh (0.075 mm) sieve. Micropowder with a specific surface area of 800–900 kg/m^2^ was selected as the material for use in this study. Portland cement with a strength grade of 42.5 was used, which conformed to the Chinese GB175-2007 standard [19]. The AES polymer composite foaming agent was selected in this paper. A certain amount of foaming agent was diluted by 60–80 times with water before being placed in the foaming machine. The diluent was mixed with the compressed air in the machine, and the foaming machine was used to form foam through the action of the compressed air.

### 2.2. Mix Design

According to the requirements in [20], the mix ratio was adjusted according to theoretical calculations and a corresponding trial mix test. The proportion of recycled foamed concrete should meet the requirements of fluidity, compressive strength, density, stability, and other indicators and should take a reasonable selection of raw materials and cement savings as the principle. The density method [21] was adopted in this test for the theoretical calculation of the mix ratio, as shown in Table 2.

Various materials were weighed according to the mixing ratios shown in Table 2; the foaming agent and water were mixed and stirred at a mass ratio of 1:60 to produce foaming liquid, and then the foaming liquid was sent to the foaming mechanism to produce the prefabricated foam required for mixing. The measured cement and MSWI powder were put into a blender for dry mixing, followed by water; then, the foamed concrete was stirred. As shown in Table 2, a quantitative amount of prefabricated foam was mixed into the paste and fully stirred. Then, the fresh foamed concrete was poured into the mold for pouring. After pouring, the paste was slightly vibrated manually to fill the mold evenly. It was not vigorously vibrated in order to prevent the foam from floating on the surface and stratify the paste. After 48 h, the mold was removed, and the test block was cured for 28 days under standard conditions.

The determination of the dry density in foamed concrete according to Chinese standard JG/T266-2011 [22]. First, a group of three test blocks were placed in a drying oven at 150 degrees Celsius and dried for more than 24 h. After the specimen was cooled to room temperature, the mass of the dried specimen was weighed to an accuracy of 1 g. The length value in each direction had to be measured at different positions. Each direction had to be measured 6 times, and the measured value had to be accurate to 1 mm. The average value of the measured length was taken as the length value in that direction, and the volume V of each foamed concrete test block was calculated. The dry density was calculated according to Equation (1). The mean dry density of 3 matching test blocks was taken as the final dry density of the test group.

(1) $$\rho_{0}=\frac{m_{0}}{V}\times 10^{6}$$

where *ρ*_0_ is the dry density of the test block in kg/m^3^; *m*_0_ is the mass of the test block after drying in g; *V* is the volume of the specimen in mm^3^.

### 2.3. Test Method

#### 2.3.1. Compressive Strength Test

The strength of the foamed concrete was tested with a pressure testing machine. After the cubic foamed concrete test block was cured for the required time, it was put into a drying box to dry. In accordance with the same steps as those in the dry density test, the dimensions of the compression test pieces were measured to calculate the compression surface area of the foamed concrete cubes. According to the steps given in the specification [20], the expected strength class was C_0.3_ to C_1_ and the pressurization rate was set to 1 kN/s. The compressive strength was calculated according to Equation (2). The compressive strength of an experimental group was the average value of the compressive strengths of three consecutive test blocks in that group.

(2) $$\sigma =\frac{F}{A}$$

where *σ* is the compressive strength of the test block in MPa; *F* is the maximum failure load of the test block in N; *A* is the compression area of the specimen in mm^2^.

#### 2.3.2. Water Absorption Test

A group of three specimens were dried in a drying oven, then removed and placed indoors in a dry, shaded area to cool [20]. After the test blocks were cooled to room temperature, their mass was weighed, giving the drying mass of the test blocks. According to the steps given in the specification [17], the test block was put into the water tank, and water was added every 24 h. The first two times that water was added, it was poured to 1/3 and 2/3 of the height of the test block, respectively; the last time to water was added, it was poured to a height that was 30 mm higher than the top of the test block. The test block was taken out and weighed after being immersed in water for 24 h, and its mass was the water absorption mass of the test block. The water absorption rate was calculated according to Equations (2) and (3), and the average water absorption rate of a group of 3 specimens was the water absorption rate of this group of test blocks.

(3) $$w=\frac{m_{g}-m_{0}}{m_{0}}\times 100$$

where *w* is the water absorption rate in %; *m*_0_ is mass of specimen after drying in g; *m_g_* is the mass of the specimen after water absorption in g.

#### 2.3.3. Impermeability Test

According to the existing test methods and specifications [17,23], the permeability of foamed concrete containing MSWI powder was evaluated according to the average penetration time of the water seepage phenomenon on the top surface of a group of six specimens. The water seepage height method was adopted in the test, and the water pressure was controlled at 0.2 ± 0.05 MPa to prevent damage to the specimen. During the test, it was observed at all times if water seepage appeared on the top surface of the specimen. When water seepage appeared on the end face of an impermeable test block, the test of the impermeable test block was immediately terminated and the time of water seepage was recorded. The time of water seepage on the top surface of each specimen was recorded when the instrument was turned off until the group of six specimens all appeared to have water seepage on the top surface. Finally, the impermeability of the foamed concrete containing MSWI powder was measured according to the average of the permeation time of the tops of all six samples. The permeation height of the sample was denoted as 150 mm.

#### 2.3.4. Testing Method of Pore Structure Parameters

According to the Chinese standard GB55008-2021, the porosity of the foamed concrete containing MSWI powder was determined with the direct mass–volume calculation method. The porosity of the foamed concrete was the percentage of the volume of pores with respect to the total volume of the foamed concrete, which directly reflected its overall compactness. The calculation is shown in Equation (4).

(4) $$\varepsilon =(1-\frac{m_{0}}{V\cdot \rho_{S}})\times 100\%$$

where is *m*_0_ is the mass of the test block after drying in kg; *ρ_S_* is the density of the foamed concrete’s base material, kg/m^3^; *V* is the volume of the foamed concrete test block (0.001 m^3^).

**Figure 2:**
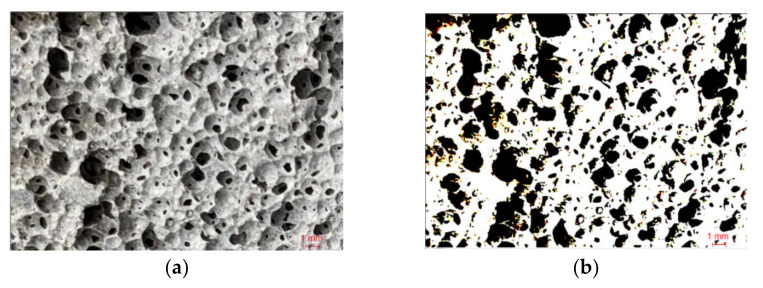
Section diagrams of the foamed concrete; (**a**) original image; (**b**) processed image.

A hacksaw was used to cut the foamed concrete test block, a relatively flat foamed concrete test block section was selected, and a brush was used to clean the section to make the section’s pores clear. The section of foamed concrete was filmed with a high-definition camera, and data on the pore parameters, such as the pore shape factor and average pore diameter, were collected after bidirectional processing, as shown in Figure 2.

**Figure 3:**
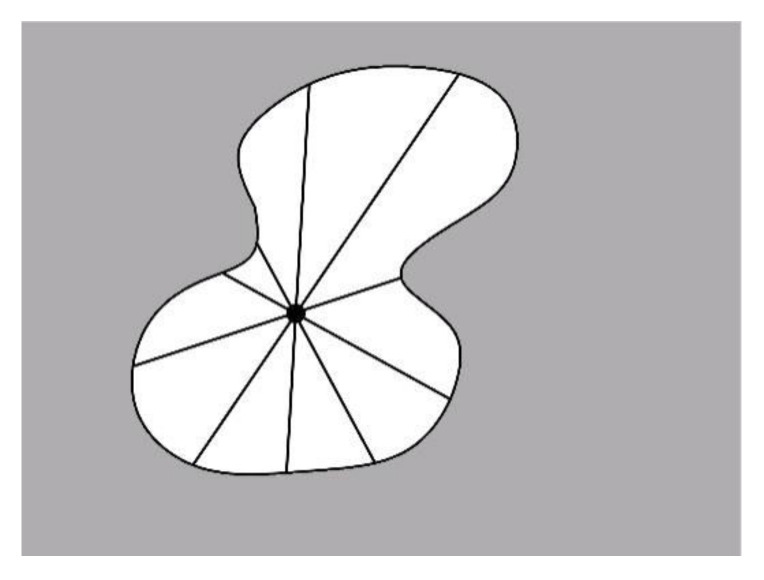
Average aperture collection method.

In order to study the change in the pore structure in more detail, quantitative parametric data on the pore structure of the foamed concrete samples containing MSWI powder were collected by software. The diameter of a single irregular pore was represented by the average of the interval between the two ends of the pore centroid, as shown in Figure 3. In this paper, the average pore diameters were divided into 0–0.2, 0.2–0.4, 0.4–0.6, 0.6–0.8, 0.8–1.0, 1.0–1.2, and above 1.2 mm as the intervals, and the proportion of the number of pores in a pore diameter interval to the total number of pores collected was taken as the distribution frequency of the pore diameter interval. The average pore size of a test group was the average of all irregular pore sizes collected from the test group of sections of the foamed concrete containing MSWI powder.

The pore shape factor was a parameter used to show the degree of pore deformation. The data collected in this paper were the pore shape factor (roundness value) in a two-dimensional plane. Equation (5) was used for the calculation; the average pore shape factor was the arithmetic average of all pore shape factors collected in this section.

(5) $$S=\frac{C^{2}}{4\pi A}$$

where *S* is the degree of stomata deviating from the circle. When *S* = 1, the shape of the stomata in a two-dimensional plane is that of a regular circle; the larger the value of *S* is, the more it deviates from a regular circle; *C* is the circumference of the projection of the pores onto a two-dimensional plane in mm; A is the area projected by the stomata onto a two-dimensional plane in mm^2^.

## 3. Results and Discussion

### 3.1. Compressive Strength of the Foamed Concrete Containing MSWI Powder

**Figure 4:**
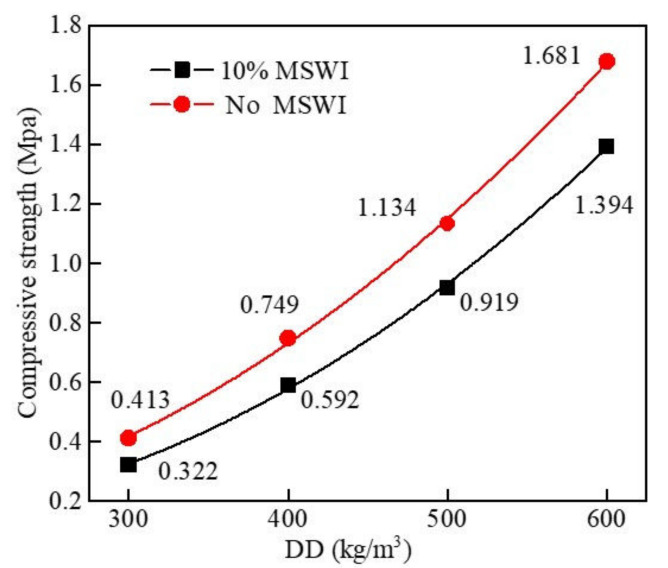
Compressive strength of foamed concrete with MSWI powder with different dry densities.

The results of the compressive strength tests on the foamed concrete with different densities are shown in Figure 4. The compressive strengths of ordinary foamed concrete and foamed concrete containing MSWI powder increased with the increase in dry density. When the dry density was 300 kg/m^3^, the compressive strength of foamed concrete containing MSWI powder was the lowest, and the compressive strength was 0.322 MPa. When the dry density was 600 kg/m^3^, the compressive strength of foamed concrete containing MSWI powder was the highest, and the compressive strength was 1.394 MPa, increasing by 332.92%. The compressive strength of foamed concrete decreased with the addition content of MSWI powder. When the dry densities of foamed concrete containing MSWI powder were 300, 400, 500, and 600 kg/m^3^, the compressive strength decreased by 22%, 21%, 19%, and 17%, respectively. The range of the decrease in compressive strength of foamed concrete with MSWI powder decreased with the increase in dry density.

A foamed concrete test block containing MSWI powder with a low dry density was prepared with a large amount of prefabricated foam, which was formed at a slower speed and increased the probability of foam damage in the paste. The compactness of the foamed concrete test block with MSWI powder after forming was low, and there were a large number of pores leading through the block and large pores, leading to a low compressive strength. As the dry density of the foamed concrete with MSWI powder increased, the foam content decreased during the preparation and the amount of cementitious material in the foamed concrete with MSWI powder increased, meaning that it could produce more hydration products. The foamed concrete test block with MSWI powder had a faster setting speed, higher early strength, and lower probability of foam damage in the paste. As a result, the structure of the foamed concrete with MSWI powder had a higher compactness, and the compressive strength was improved. The reason for why the compressive strength decreased was that the activity of the MSWI powder was not high and the pozzolanic effect was weak, leading to a decline in the compressive strength of recycled foamed concrete with MSWI powder [24].

**Figure 5:**
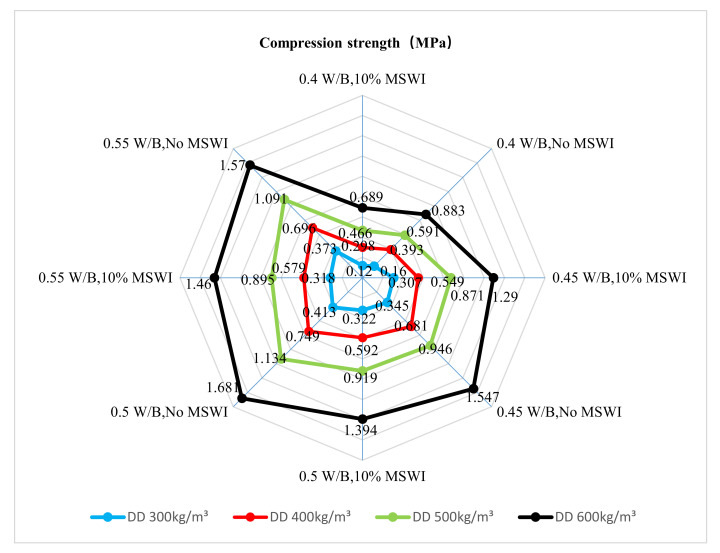
Compressive strength of recycled foamed concrete with MSWI powder with different w/c ratios.

Figure 5 shows the rule of the changes in the dry density of foamed concrete with different w/c ratios when the MSWI powder substitution content was 10%. When the content of MSWI powder was 10% and the dry density was between 300 and 500 kg/m^3^, the compressive strength of the foamed concrete containing MSWI powder first increased and then decreased with the increase in the w/c ratio. When the w/c ratio of the foamed concrete with MSWI powder was 0.5, its compressive strength reached the maximum value. Compared with ordinary foamed concrete, the compressive strength of the foamed concrete with MSWI powder could be reduced by adding MSWI powder. When the dry density of the foamed concrete with MSWI powder was 600 kg/m^3^ and the w/c ratio was 0.55, its compressive strength was not different from that of traditional, ordinary concrete.

When the w/c ratio of the foamed concrete with MSWI powder was small, there was not enough free water in the paste, and the paste could only absorb the water required for the hydration reaction during the initial setting process. The loss of water and the rupture led to an increase in the number of large-sized pores in the structure, and the reduction of the compactness of the paste structure reduced the compressive strength. When the w/c ratio increased, the fluidity of the paste increased and the friction between the foam and the material decreased, making the foam difficult to burst. The foamed concrete specimens with MSWI powder became denser and their compressive strength increased after solidification and hardening. When the w/c ratio exceeded 0.50, in the curing process of the foamed concrete with MSWI powder, the phenomenon of the bleeding of paste began to occur, and the excess free water in the paste absorbed the heat generated by the hydration reaction and evaporated, leaving a large number of capillary pores and cracks [25].

The internal structural compactness of the degraded foamed concrete decreased, resulting in a decline in compressive strength. When the dry density was 600 kg/m^3^ and the w/c ratio increased from 0.4 to 0.55, the compressive strength of the foamed concrete with MSWI powder increased with the increasing w/c ratio because the particle size of the MSWI powder was smaller than that of cement. When replacing equal-quality cements, more cementitious materials could be introduced, while the MSWI powder had a morphological effect, which could make the paste uniform. The MSWI powder had a micro-aggregate effect and an effect on the activity [26,27]. When the w/c ratio was high, the effect on the activity was obvious and more hydration products could be produced. The foamed concrete with MSWI powder became denser after forming, and the compressive strength increased.

**Figure 6:**
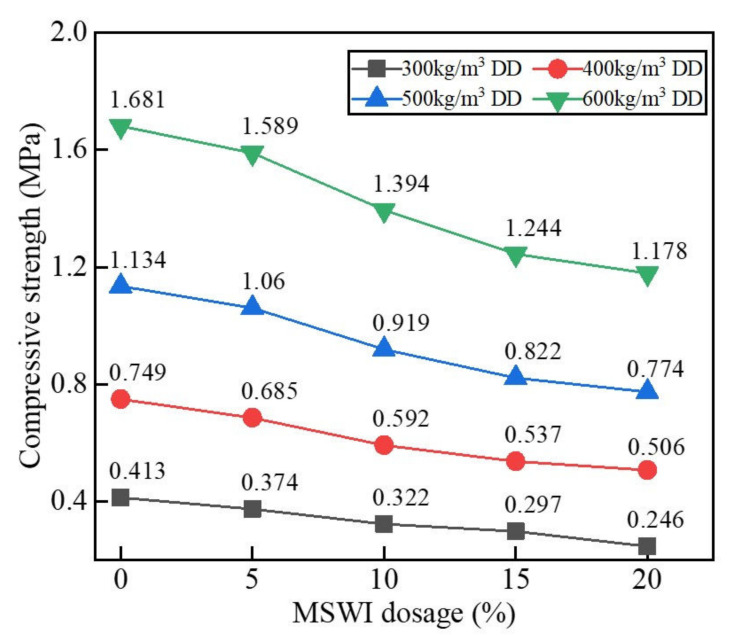
Compressive strength of the recycled foamed concrete with different MSWI micro-powder contents.

The results of the compressive strength test on the foamed concrete with different MSWI powder contents at a w/c ratio of 0.5 are shown in Figure 6. When the w/c ratio was 0.5 and the dry density was constant, the compressive strength of the foamed concrete with MSWI powder decreased with the increase in the substitution with MSWI powder. According to Table 3, when the amount of substitution with reclaimed MSWI powder reached 20%, the minimum compressive strength of the foamed concrete test block with reclaimed MSWI powder and with a dry density of 300 kg/m^3^ was less than 0.225 MPa, which did not meet the requirements of the specification [20].

When the density of the foamed concrete with MSWI powder was low, there was less cementing material in the paste, and the amount of foam was greater. With the increase in MSWI powder content, the proportion of cement in the cementing material decreased, and the strength of the foamed concrete decreased due to hydration products. At the same time, the ultra-fine MSWI powder made after crushing and grinding had certain activated ingredients, but the overall activity was not high and the early pozzolanic effect was weak, leading to a decline in the compressive strength of the foamed concrete with MSWI powder. In addition, when the content of MSWI powder increased to a certain extent, although it had a morphological effect and could make the paste more uniform, the reduction in hydration products caused the foamed concrete with MSWI powder to be unable to completely wrap the foam in the hardening process. The probability of the foam bursting in the concrete structure became higher, and more pores going through the structure were formed [28].The internal structural deterioration of the foamed concrete with MSWI powder resulted in a decrease in the compressive strength.

### 3.2. Water Absorption Rate of the Foamed Concrete Containing MSWI Powder

**Figure 7:**
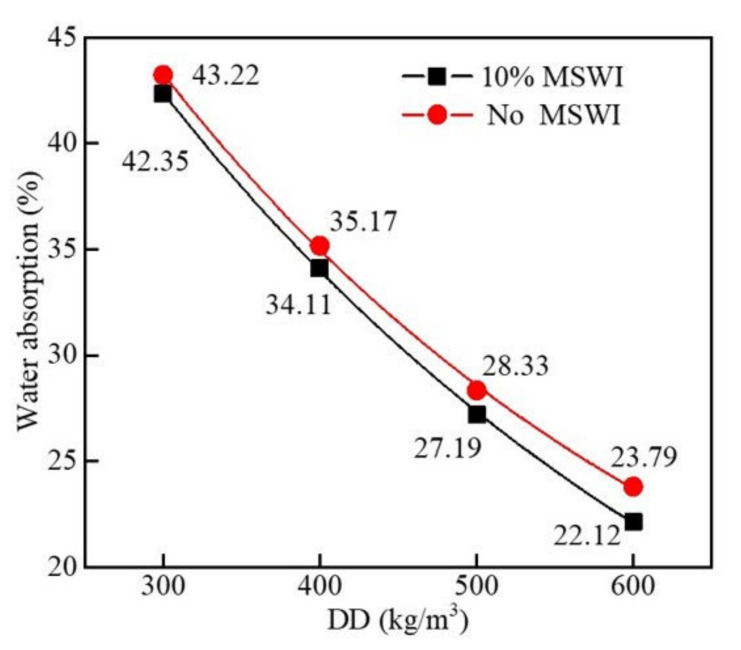
Water absorption of the recycled foamed concrete containing MSWI powder with different dry densities.

Figure 7 shows the results of the water absorption test of the foamed concrete containing MSWI powder with different dry densities when the w/c ratio was 0.5 and the MSWI powder content was 10%. As can be seen in Figure 7, the water absorption of the foamed concrete decreased with the increase in dry density. When the dry density of the foamed concrete with MSWI powder was 300 kg/m^3^, the water absorption of the sample was the highest, and the value was 42.55%. When the dry density was increased to 600 kg/m^3^, the water absorption rate was the lowest, and the value was 23.79%. The decrease in the water absorption of the foamed concrete with MSWI powder decreased with the increase in dry density. When the dry density of the foamed concrete with MSWI powder was 300, 400, 500, and 600 kg/m^3^, respectively, the water absorption rate of the foamed concrete with MSWI powder decreased by 2.01%, 3.01%, 4.02%, and 7.02%. Through a comparison with ordinary foamed concrete, MSWI powder was found to reduce water absorption, but the effect was limited. On the one hand, the foamed concrete containing MSWI powder with a low density had many internal pores, and there were a large number of connecting pores and large pores, which increased the water absorption. With the increase in dry density, the number of through-pores in the foamed concrete and, especially, the number of large pores decreased, resulting in a decrease in water absorption. On the other hand, when preparing the foamed concrete test blocks with a low dry density, the amount of foam increased, resulting in more free water being introduced by the foam. The decrease in the amount of cementitious material caused the foamed concrete to bleed water in the hardening process, leading to a reduction in the structural compactness and even the paste stratification of the foamed concrete, causing a large number of connected pores to be formed and increasing the water absorption rate [29].

**Figure 8:**
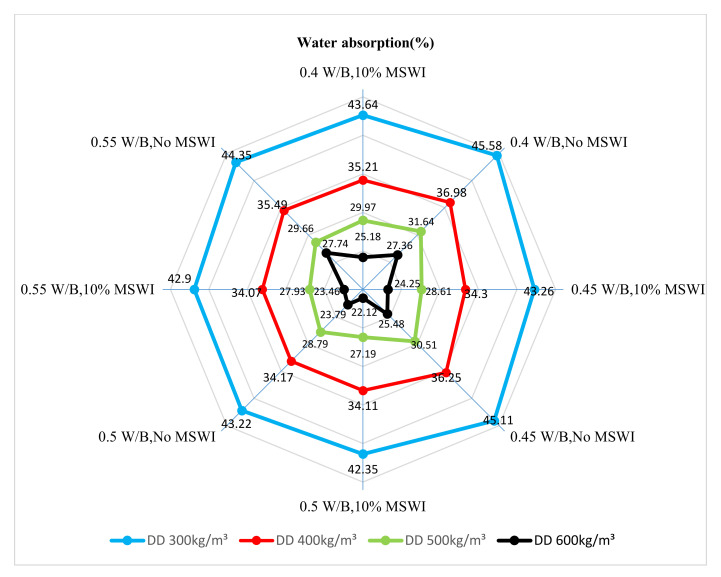
Water absorption of the recycled foamed concrete containing MSWI powder with different w/c ratios.

Figure 8 shows the water absorption of the foamed concrete with MSWI powder at different w/c ratios. When the MSWI powder content was 10%, the water absorption of the foamed concrete first decreased and then increased with the increase in the w/c ratio. When the w/c ratios were 0.4 and 0.5, the water absorption of the foamed concrete with MSWI powder reached the maximum and minimum values, respectively. Through a comparison with ordinary foamed concrete, MSWI powder was shown to reduce the water absorption of the foamed concrete, which improved its water tightness. When the w/c ratio was low, the paste’s consistency was high, and a large number of cementitious materials condensed into groups during the stirring process, resulting in an uneven particle distribution. As the friction between particles and foam increased, it was easy for foam deformation and even the bursting of bubbles to occur. The increasing number of through-pores in the foamed concrete with MSWI powder reduced the compactness and increased the water absorption. In addition, there was not enough free water in the paste with a low w/c ratio, so, in the initial setting process of the foamed concrete, the cementing material could only absorb the water needed for the hydration reaction from the foam mixed with the cement paste [30].

**Figure 9:**
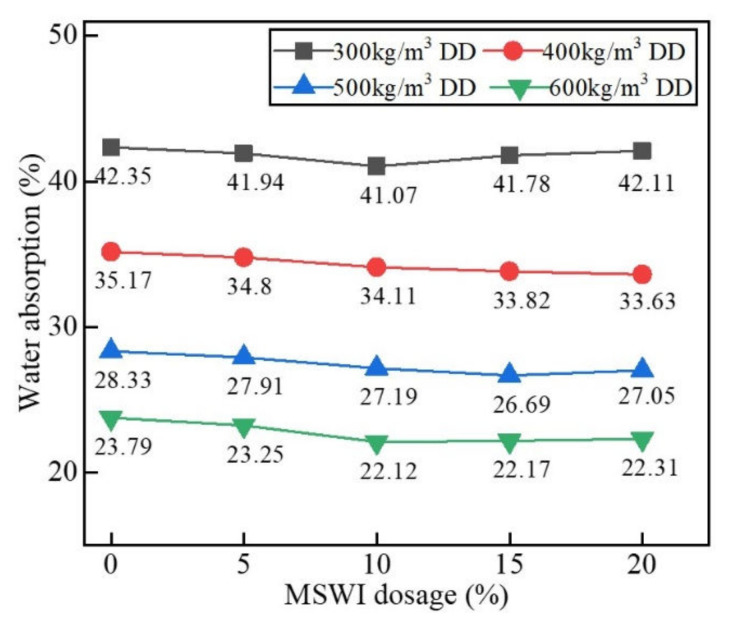
Water absorption rate of the recycled foamed concrete containing MSWI powder with different dosages.

Figure 9 shows the results of the test of water absorption of the recycled foamed concrete with different MSWI powder contents at a w/c ratio of 0.5. No matter how the dry density changed, the water absorption of the foamed concrete decreased with the increase in MSWI powder content, but the decrease was small. This was because the foamed concrete with MSWI powder had good water retention, which could prevent the base paste from bleeding and stratification after pouring. In the hardening process, the MSWI powder also had an active effect, as it could react with the hydration products in the foamed concrete in a secondary hydration reaction so as to make the foamed concrete test block denser and play a role in regulating the pores and reducing porosity. The replacement of cement with MSWI powder also reduced the heat of hydration, resulting in less water evaporation and fewer through-pores [8].

### 3.3. Results of the Impermeability Test of the Foamed Concrete Containing NSWI

**Figure 10:**
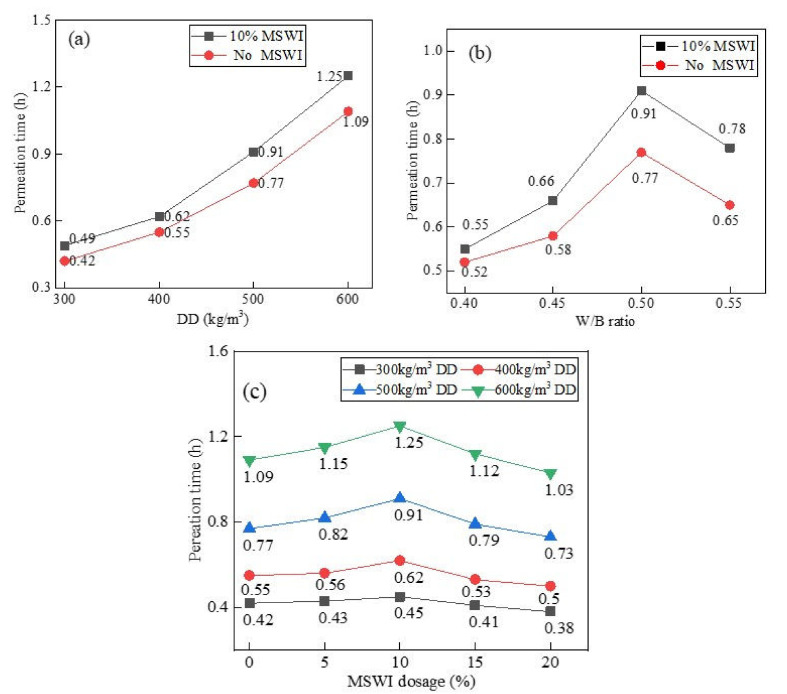
Impermeability of the recycled foamed concrete containing MSWI powder with different dry densities (**a**), W/B ratios (**b**), and MSWI powder dosages (**c**).

Figure 10a shows the results of the impermeability test of the foamed concrete containing MSWI powder with different dry densities when the w/c ratio was 0.5 and the MSWI content was 10%. With the increase in the dry density of the foamed concrete, the permeability time was prolonged and the impermeability was improved. When the dry density increased to 600 kg/m^3^, the foamed concrete with MSWI powder had the best impermeability, and the penetration time was 1.25 h. The MSWI powder could effectively improve the impermeability of the foamed concrete. When the dry densities were 300, 400, 500, and 600 kg/m^3^, the impermeability increased by about 16.7%, 12.7%, 18.1%, and 14.7%. Under the condition of a constant w/c ratio, foamed concrete containing MSWI powder with a low dry density was prepared by adding a large number of prefabricated foams; the reduction of gelled particles led to a slow hydration reaction in the early stage, and the foam in the paste was easy to burst. However, there were more pores in the test block, and the overall density of the structure was low. Under the action of external pressure, water could enter the interior of foamed concrete along the through-pores, resulting in poor impermeability [31].

Figure 10b shows the results of the impermeability test of foamed concrete with different w/c ratios when the dry density was 500 kg/m^3^ and the MSWI powder content was 10%. With the increase in the w/c ratio, the infiltration time of the foamed concrete containing MSWI powder showed a trend of first extending and then shortening. When the w/c ratio was 0.4, the permeability time was 0.52 h, which was the shortest permeability time and the worst permeability performance. When the w/c ratio was 0.5, the infiltration time was 0.77 h, the infiltration time was the longest, and the anti-permeability performance was the best. When the w/c ratio was more than 0.5, the penetration time was shortened and the anti-permeability decreased. Compared with ordinary foamed concrete, the addition of MSWI powder could slightly improve the impermeability of the recycled foamed concrete. When the w/c ratio was 0.4–0.55, the impermeability increased by 6%, 13.8%, 18.1%, and 20%, respectively.

The results of the impermeability test of the foamed concrete containing MSWI powder with different contents are shown in Figure 10c. The penetration time of the foamed concrete first increased and then decreased with the increase in MSWI powder content, and this trend became more obvious with the increase in dry density [32]. When the dry density was 600 kg/m^3^ and the MSWI powder content was 10%, the impermeability of the recycled foamed concrete was the best, and the permeation time was 1.25 h. The addition of MSWI powder could improve the impermeability of the foamed concrete. The MSWI powder had a good morphological effect, which could improve the fluidity of the foamed concrete and prevent the separation of fresh paste and rupturing of the foam. Secondly, the MSWI powder resulted in hydration activity, which could accelerate the hydration reaction in the cement and prevent foam deformation and rupture. Finally, the particle size of the MSWI powder was smaller than that of cement, and it could fill through-pores and cracks in the foamed concrete, thus playing a role in regulating pores. However, due to the limited activity and the delay of the MSWI powder, the improvement of the anti-seepage performance was limited. With the further increase in MSWI powder content, the proportion of cement in the foamed concrete paste decreased, and the production of hydration products decreased, leading to the deterioration of the internal structure and a decrease in the compactness. Under the action of external pressure, water was more likely to enter the recycled foamed concrete test block, resulting in poor impermeability [33].

### 3.4. Pore Structural Analysis of the Foamed Concrete Containing MSWI Powder

**Figure 11:**
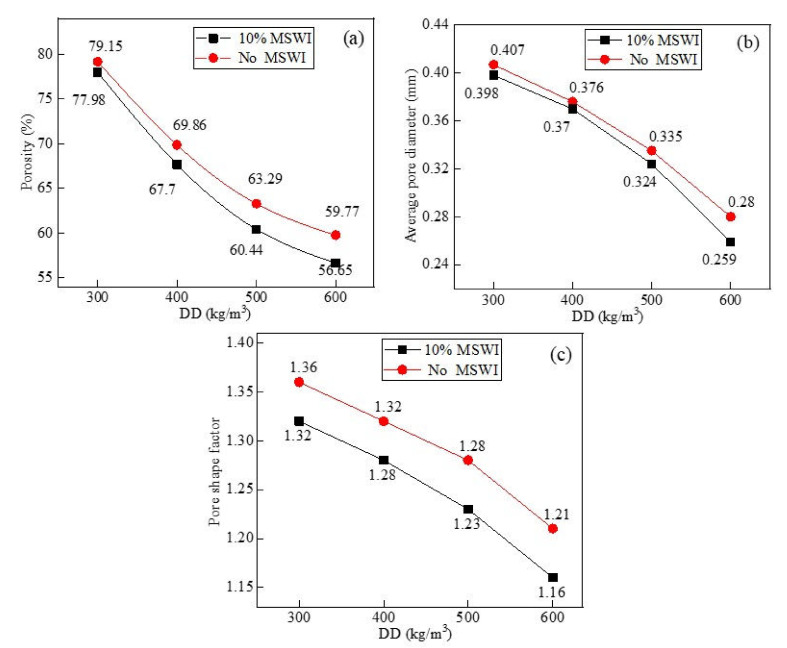
Porosity (**a**), average pore diameter (**b**), and pore shape factor (**c**) of the recycled foamed concrete containing MSWI powder with different dry densities.

Figure 11 shows the results for the porosity, average pore size, and pore shape factor of the foamed concrete at different dry densities. As can be seen from Figure 11a, the porosity of the foamed concrete containing MSWI powder decreased with the increase in its dry density. When the dry density was 300 kg/m^3^, the porosity of the foamed concrete was 77.98%. When the dry density was 600 kg/m^3^, the porosity of the foamed concrete was the lowest, which was 56.65%, decreasing by 21.33%. The porosity of the foamed concrete decreased with the addition of MSWI powder. When the dry density was 300, 400, 500, and 600 kg/m^3^, the porosity decreased by about 1.17%, 2.16%, 2.85%, and 3.12%. As can be seen from Table 4, when the MSWI powder content was 10% and the w/c ratio was 0.5, the increase in the dry density of the foamed concrete reduced the distribution range of the internal pore size. The distribution frequency in the large-size interval (above 0.8 mm) decreased, while the number of small pores in the interior increased. As can be seen in Figure 11b, with the increase in the dry density of the foamed concrete, the average pore size decreased. When the dry density of the foamed concrete containing MSWI powder was 300 kg/m^3^, the average pore diameter was 0.398 mm. However, when the dry density of the MSWI powder foams was increased to 600 kg/m^3^, the average pore size was 0.259 mm, which was a decrease of about 34.9%. It can be seen in Figure 11c that, with the increase in the dry density of the foamed concrete, the average pore shape factor decreased and approached 1. Through a comparison with ordinary foamed concrete, the addition of MSWI powder was shown to reduce the average pore shape factor.

**Figure 12:**
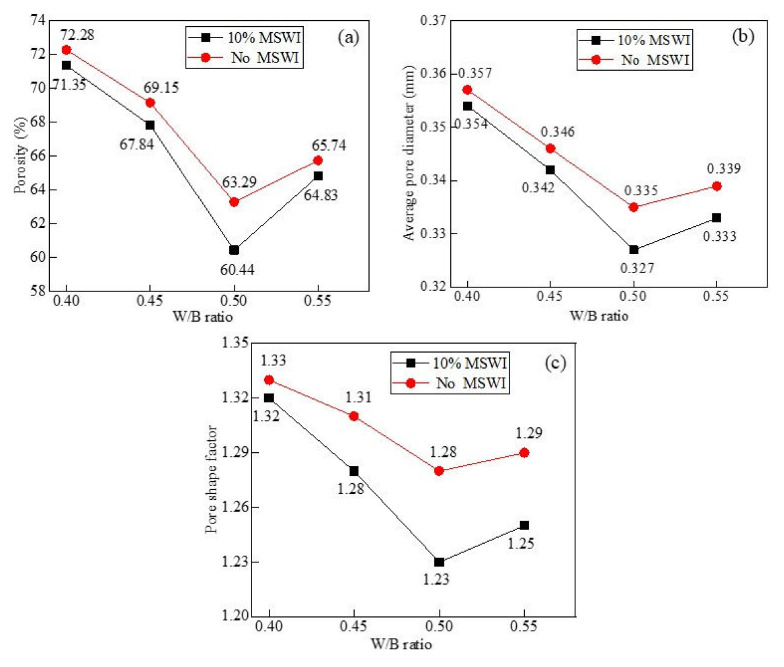
Porosity (**a**), average pore diameter (**b**), and pore shape factor (**c**) of the recycled foamed concrete containing MSWI powder with different water–binder ratios.

The pore structures of the foamed concrete containing MSWI powder with different w/c ratios are shown in Table 5 and Figure 12. As can be seen in Table 5, when the w/c ratio was 0.4, only 80% of the pores were distributed within the range of 0−0.4 mm, and there were many large pores in the foamed concrete containing MSWI powder. When the w/c ratio was 0.45, the distribution frequency of the pore diameter increased in the ranges of 0−0.2, 0.2−0.4, and 0.4−0.6 mm, and the pore size in the foamed concrete containing MSWI powder decreased, resulting in a narrower pore diameter distribution range. When the w/c ratio was 0.5, the pore size in the range of 0−0.2 mm accounted for the highest proportion of 66.4%. When the w/c ratio was 0.55, the distribution frequencies of the pore sizes in the 0−0.2 and 0.2−0.4 mm ranges decreased, and those in the ranges of 0.6−0.6, 0.8−1.0, and above 1.0 mm increased. At this time, the pore size inside the foamed concrete containing MSWI powder increased, resulting in a wider pore size distribution range.

As can be seen in Figure 12a, under the condition of a dry density of 500 kg/m^3^ and MSWI powder content of 10%, with the increase in the w/c ratio, the pores of the foamed concrete containing MSWI powder first decreased and then increased. When the w/c ratio was 0.4, the internal porosity of the foamed concrete containing MSWI powder was the highest, which was 71.35%. When the w/c ratio was 0.5, the porosity of the foamed concrete containing MSWI powder was the lowest, which was 60.44%, decreasing by about 10.91%. As can be seen in Figure 12b, when the dry density was 500 kg/m^3^ and the MSWI powder content was 10%, the average pore size of the foamed concrete containing MSWI powder first decreased and then increased, but the range of variation was different. When the w/c ratio was 0.5, the average pore size of the foamed concrete containing MSWI powder was the smallest (0.327 mm), which was decrease of about 7.7% compared with that when the w/c ratio was 0.4. Through a comparison with ordinary foamed concrete, the addition of MSWI powder was shown to adjust the pore structure of the foamed concrete and reduce the average pore size inside it. As can be seen in Figure 12c, with the increase in the w/c ratio, the average pore shape factor of the foamed concrete containing MSWI powder first decreased and then increased. In a comparison with ordinary foamed concrete, the addition of MSWI powder was shown to adjust the pore structure of the foamed concrete, reduce its average pore shape factor, and reduce the degree of pore deformation. It can be seen from the above results that the incorporation of MSWI powder could regulate the porosity, average pore size, and pore shape factor of the foamed concrete [34,35,36].

**Figure 13:**
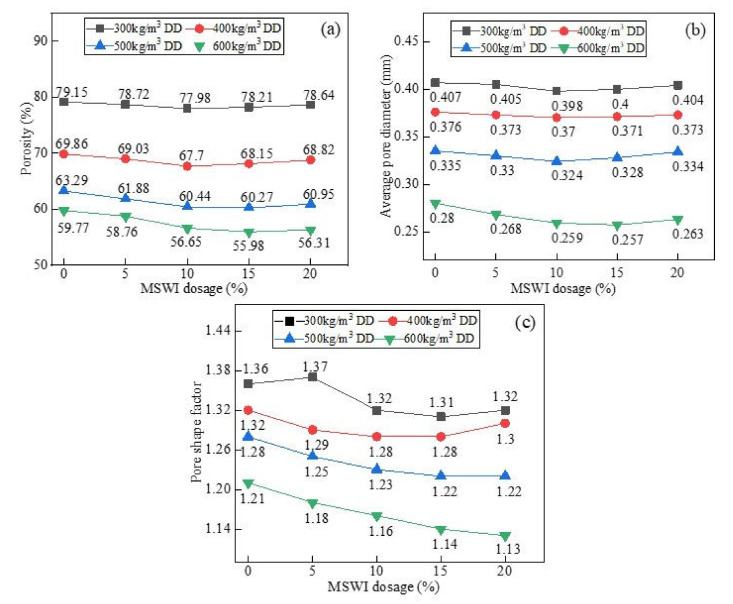
Porosity (**a**), average pore diameter (**b**), and pore shape factor (**c**) of the recycled foamed concrete with different MSWI powder contents.

As can be seen in Table 6, when the dry density was 500 kg/m^3^ and the w/c ratio was 0.5, the pore size distribution range of the foamed concrete containing MSWI powder first decreased and then expanded with the increase in MSWI powder content. These results showed that the number of small-size pores formed in the foamed concrete first increased and then decreased, while the number of large-size pores first decreased and then increased. When the MSWI powder content was 10%, the pore size distribution range was the smallest, and the number of pores in the range of 0–0.4 mm accounted for 90.3% of the total number of pores collected. It can be seen in Figure 13a that, when the dry density of the foamed concrete containing MSWI powder was low and the w/c ratio remained unchanged, the porosity of the foamed concrete slightly changed with the increase in MSWI powder content. However, when the dry density of the foamed concrete containing MSWI powder was high, the porosity of the foamed concrete decreased with the increase in MSWI powder content under the condition of a constant w/c ratio. As can be seen in Figure 13b, when the w/c ratio of the foamed concrete containing MSWI powder remained unchanged, the average pore size of the foamed concrete first decreased and then increased with the increase in MSWI powder content. When the content was 10%, the average pore size of the foamed concrete containing MSWI powder was the smallest. It can be seen in Figure 13c that the addition of MSWI powder reduced the degree of stomatal deformation, but the degree of the decrease was not significant.

The addition of MSWI powder was able to adjust the internal pore structure of the foamed concrete. Because the MSWI powder had a good morphological effect that could prevent the stratified segregation of the freshly mixed paste and the deformation and rupture of the internal foam, the degree of deformation of the pores was reduced. Secondly, the MSWI powder resulted in hydration activity, and the effect of the activity could generate more hydration products, which accelerated the hydration reaction in the cement and optimized the internal pore structure of the foamed concrete. The particle size of the MSWI powder was smaller than that of the cement. The MSWI powder that had not undergone a hydration reaction after being mixed into the foamed concrete could fill the pores inside it. This made the interior of the foamed concrete structure denser, and the porosity and the size of the pores were reduced. However, due to the low activity and retardation of the MSWI powder, the effect of the adjustment of the cellular structure of the foamed concrete was limited. The addition of MSWI powder at a low dosage reduced the proportion of cement in the cementitious material, reducing the heat of hydration released during curing. The evaporation of free water inside the paste was reduced, and the probability of the formation of large-sized pores and through-holes was reduced. When the content of the MSWI micropowder was higher, the proportion of cement in the foamed concrete paste decreased, and the generation of hydration products decreased. The deterioration of the internal structure of the foamed concrete resulted in an increase in the porosity of the foamed concrete and an increase in the internal large-sized pores [37,38].

### 3.5. The Relationship between Pore Structural Parameters and Macroscopic Parameters

**Figure 14:**
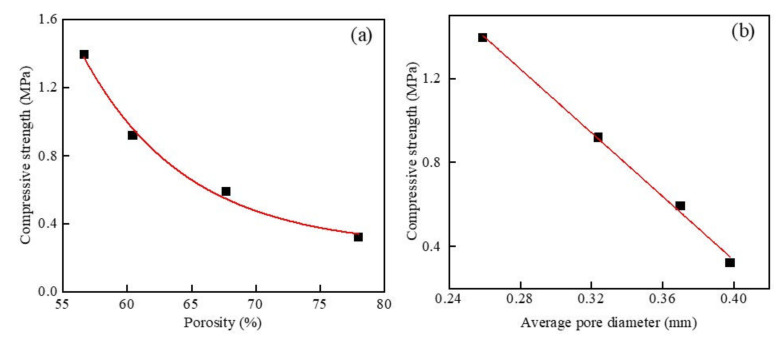
Compressive strength versus porosity (**a**) and average pore diameter of (**b**) the recycled foamed concrete containing MSWI powder with different densities.

Figure 14 shows the results of the compressive strength as a function of porosity and mean pore size at different dry densities. It can be seen in Figure 14a that, when the w/c ratio was 0.5 and the MSWI powder content was 10%, with the increase in the porosity of the foamed concrete containing MSWI powder, its compressive strength decreased. After the combination, the relationship between the two was y = 1243.654e^−0.1238x^ + 0.26244, which was an exponential function relationship, and the correlation coefficient was R^2^ = 0.993. It can be seen in Figure 14b that, when the w/c ratio was 0.5 and the content of MSWI powder was 10%, as the average pore size of the foamed concrete containing MSWI powder increased, its compressive strength decreased. After fitting, the following relationship between the two was obtained: y = −7.5873x + 3.36939; the correlation coefficient R^2^ reached 0.997. By comparing the results of the above analysis, it can be seen that, when only the dry density changed, the compressive strength of the foamed concrete containing MSWI powder was mainly affected by the average pore size of the pores in it. This was because, with the increase in dry density, the number of pores in the foamed concrete containing MSWI powder decreased, especially the number of large pores. The rapid reduction of the average pore size of the internal pores of the foamed concrete containing MSWI powder made the structural compactness increase continuously, so the compressive strength was significantly improved.

**Figure 15:**
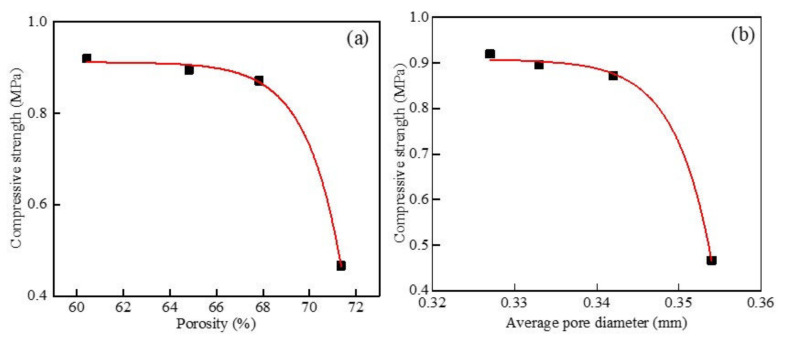
Compressive strength versus porosity (**a**) and average pore size (**b**) of the recycled foamed concrete containing MSWI powder with different w/c ratios.

When the dry density was 500 kg/m^3^ and the powder content was 10%, the foamed concrete containing MSWI powder with different w/c ratios was analyzed, and the results are shown in Figure 15. When the dry density was 500 kg/m^3^ and the content of the recycled fine MSWI powder was 10%, with the increase in the porosity of the foamed concrete containing MSWI powder, the compressive strength of the foamed concrete containing MSWI powder decreased. After data fitting, the following relationship between the two was obtained: y = −1.749619 × 10^−21^ × e^0.65856x^ + 0.9118, showing an exponential relationship, similarly to the Ryshkevitch model [38]; the correlation coefficient was R^2^ = 0.998. As can be seen in Figure 15b, when the dry density was 500 kg/m^3^ and the MSWI powder content was 10%, the compressive strength of the foamed concrete containing MSWI powder decreased with the increase in the average pore size. After data fitting, the following relationship between the two was obtained: y = −5.016 × 10^−35^ × e^220.75x^ + 0.90735, which was an exponential relationship, and the correlation coefficient was R^2^ = 0.998. When only the w/c ratio changed, the compressive strength of the foamed concrete containing MSWI powder was significantly affected by the porosity. This was mainly because the porosity of the foamed concrete changed more than the average pore size with the change in the w/c ratio. The numbers of large-sized pores and through-pores in the foamed concrete containing MSWI powder varied greatly, which affected its compactness. The compactness of the foamed concrete containing MSWI powder was directly related to its compressive strength, so there was a more obvious effect on its compressive strength.

**Figure 16:**
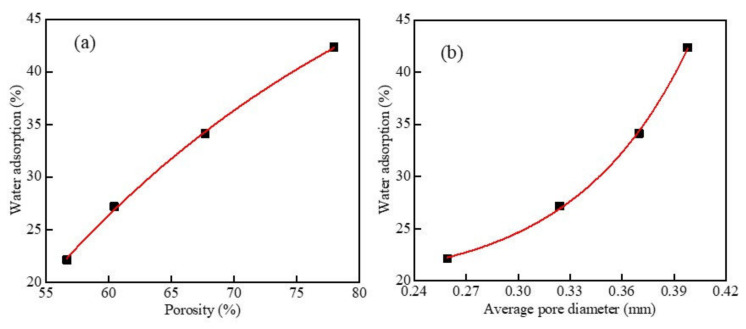
Water absorption versus porosity (**a**) and average pore diameter (**b**) of the recycled foamed concrete containing MSWI powder with different dry densities.

Figure 16 shows the results of water absorption as a function of porosity and mean pore size at different dry densities. When the w/c ratio was 0.5 and the content of MSWI powder was 10%, the water absorption increased with the increase in the porosity of the foamed concrete containing MSWI powder. This showed that the number of through-pores inside the sample accounted for the greater proportion of the total number of pores at this time. After fitting, the relationship between the two was obtained as y = −246.335e^−0.0318x^ + 62.92733, which was an exponential function, and the correlation coefficient was R^2^ = 0.999. It can be seen in Figure 16b that, when the w/c ratio was 0.5 and the MSWI powder content was 10%, the water absorption of the foamed concrete increased with the increase in the average pore size. After fitting, the relationship between the two was y = 0.0562e^15.0921x^ + 19.4323, which was an exponential function, and the correlation coefficient was R^2^ = 0.999. When only the dry density changed, the water absorption of the foamed concrete containing MSWI powder was significantly affected by the porosity. This was because the compactness of the foamed concrete containing MSWI powder increased with the increase in dry density, while the porosity of the internal pores changed more obviously compared with the average pore size. With the increase in dry density, the number of large-sized pores and through-pores in the foamed concrete containing MSWI powder decreased remarkably. The water absorption rate could directly reflect the proportion of through-pores in all pores in the foamed concrete containing MSWI powder. The larger the proportion of through-pores, the higher the water absorption rate of the foamed concrete containing MSWI powder. Therefore, the water absorption of the recycled foamed concrete containing MSWI powder was obviously affected by its porosity.

**Figure 17:**
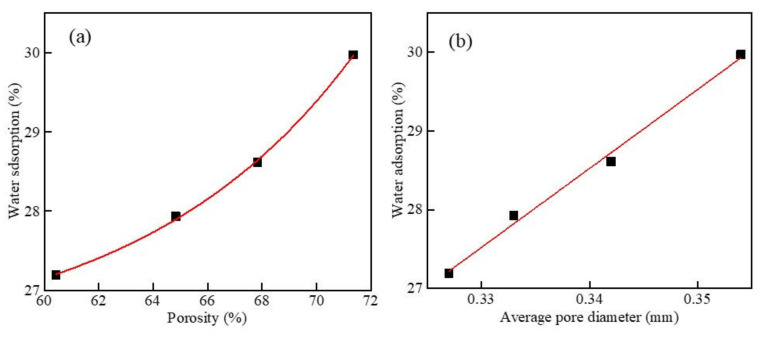
Water absorption versus porosity (**a**) and average pore diameter (**b**) of the recycled foamed concrete containing MSWI powder with different water–binder ratios.

Figure 17 shows the results of water absorption as a function of porosity and mean pore size at different w/c ratios. It can be seen in Figure 17a that, when only the w/c ratio of the foamed concrete containing MSWI powder changed, with the increase in the porosity of the foamed concrete containing MSWI powder, its water absorption increased. The relational formula was y = 4.5612 × 10^−4^e^0.1261x^ + 26.2701, which was an exponential function; the increase was from slow to fast, and the correlation coefficient was R^2^ = 0.998. When the porosity was greater than 68%, the water absorption rate rapidly increased with the increase in the porosity. At this time, most of the pores in the foamed concrete were through-pores, and the increase in the proportion of through-pores led to an increase in the water absorption rate. It can be seen in Figure 17b that, when the dry density was 500 kg/m^3^, the content of MSWI powder was 10%, and only the w/c ratio changed. With the increase in the average pore size of the foamed concrete containing MSWI powder, its water absorption increased linearly, and the rate of increase was relatively stable. At this time, the relationship between the average pore size and water absorption was y = 100.2899x − 5.57326, and the correlation coefficient was R^2^ = 0.993. By comparing the results of the above analysis, it can be seen that, when only the w/c ratio changed, the water absorption of the foamed concrete containing MSWI powder was significantly affected by the porosity. With the change in the w/c ratio, the porosity of the foamed concrete containing MSWI powder changed more obviously than the average pore size, and the number of large-sized pores and through-pores in the foamed concrete containing MSWI powder changed greatly. The water absorption rate could directly reflect the proportion of through-pores to all pores in the foamed concrete containing MSWI powder. The larger the proportion of through-hole pores, the higher the water absorption rate. Therefore, the water absorption of the foamed concrete containing MSWI powder was affected more by porosity.

**Figure 18:**
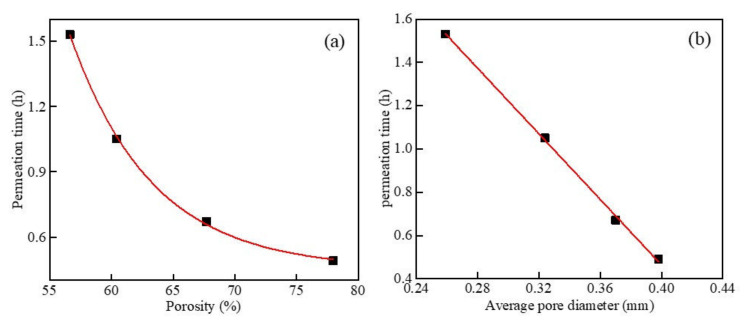
Impermeability versus porosity (**a**) and average pore diameter (**b**) of the recycled foamed concrete containing MSWI powder with different dry densities.

Figure 18 shows the results of impermeability as a function of porosity and mean pore size at different dry densities. It can be seen in Figure 18a that, when only the dry density changed, the impermeability of the foamed concrete containing MSWI powder decreased with the increase in the porosity. After fitting, the relationship between the two was y = 5352.578 e^−0.1503x^ + 0.453, which was an exponential function, and the correlation coefficient was R^2^ = 0.999. It can be seen in Figure 18b that, when the w/c ratio was 0.5 and the content of MSWI powder was 10%, with the change in dry density, the impermeability of the foamed concrete containing MSWI powder decreased with the increase in average pore size. After fitting, the relationship between the two was y = −7.57589x + 3.49376, which was linearly correlated, and the correlation coefficient was R^2^ = 0.998. By comparing the results of the above analysis, it can be seen that, when only the dry density changed, the impermeability of the foamed concrete containing MSWI powder was significantly affected by the porosity. With the increase in dry density, the compactness of the recycled foamed concrete containing MSWI powder increased continuously, while the porosity of the internal pores changed more than the average pore size. With the increase in dry density, the numbers of large-sized pores and through-pores in the foamed concrete containing MSWI powder were greatly reduced. The impermeability of the foamed concrete containing MSWI powder was related to the proportion of internal through-pores. The lower the proportion of through-pores, the better the impermeability of the foamed concrete containing MSWI powder. Therefore, the impermeability of the foamed concrete containing MSWI powder was obviously affected by the porosity.

**Figure 19:**
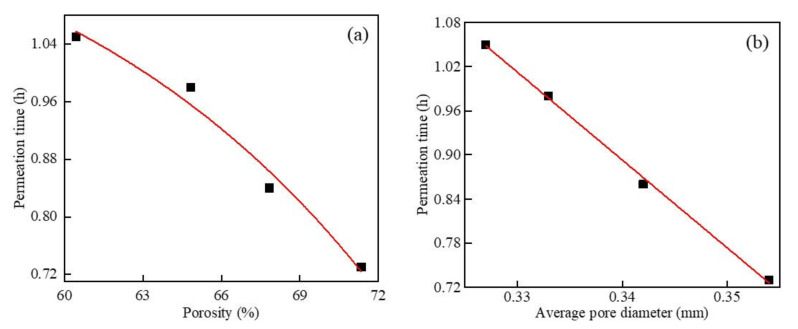
Impermeability versus porosity (**a**) and average pore diameter (**b**) of the recycled foamed concrete containing MSWI powder with different water–binder ratios.

Figure 19 shows the results of impermeability as a function of porosity and mean pore size at different w/c ratios. It can be seen in Figure 19 that, when only the w/c ratio changed, the impermeability of the foamed concrete containing MSWI powder deteriorated with the increase in the porosity. After fitting, the relationship between the two was an exponential function (y = −0.00238e^0.07718x^ + 1.3097), and the correlation coefficient was R^2^ = 0.980. It can be seen in Figure 19b that, when the dry density was 500 kg/m^3^, the content of fine MSWI powder was 10%, and only the w/c ratio changed, the impermeability became worse with the increase in the average pore size of the foamed concrete. After fitting, the relationship between the two was y = −11.9565x + 4.9583, which was linearly correlated, and the correlation coefficient was R^2^ = 0.998. By comparing the results of the above analysis, it can be seen that when only the w/c ratio changed, the impermeability of the foamed concrete containing MSWI powder was mainly affected by the porosity. With the change in the w/c ratio, the porosity of the recycled foamed concrete containing MSWI powder changed more obviously than the average pore size, and the number of large-sized pores and through-pores in the foamed concrete containing MSWI powder changed significantly. The impermeability of the foamed concrete containing MSWI powder was related to the proportion of internal through-holes. The lower the proportion of through-holes, the better the impermeability of the foamed concrete containing MSWI powder. Therefore, the impermeability of the recycled foamed concrete containing MSWI powder was obviously affected by the porosity.

## 4. Conclusions

In this paper, the effects of the dry density, water–cement ratio, and MSWI powder content on the performance and pore structure of foamed concrete containing MSWI powder were studied with a single-factor mix ratio test, and the following conclusions were obtained:

(1) The compressive strength of the foamed concrete containing MSWI powder increased with the increase in dry density, while the compressive strength first increased and then decreased when the w/c ratio increased. The hydration activity of the MSWI powder was low, and the incorporation of MSWI powder reduced the proportion of cement in the foamed concrete slurry, thus reducing the amounts of hydration products. During the hardening process, the probability of foam bursting increased, resulting in the formation of more through-pores and the reduction of compressive strength. When the MSWI powder content was 10% and the dry density was 600 kg/m^3^, the compressive strength of the foamed concrete increased with the increase in the w/c ratio, and the optimal MSWI powder content was 10%.
(2) With the increase in dry density, the water absorption rate decreased, the infiltration time was prolonged, and the impermeability was improved. The water absorption of the foamed concrete first increased and then decreased with an increasing w/c ratio. When the dry density was 500 kg/m^3^ and the MSWI powder content was 10%, the impermeability of the foamed concrete with a w/c ratio of 0.5 was the best. The MSWI powder had an active effect and a micro-aggregate effect, which could effectively fill capillaries and through-pores and optimize the internal pore structure of the foamed concrete, thus improving its impermeability.
(3) With the increase in dry density, the porosity and average pore shape coefficient of the foamed concrete decreased. The characteristic parameters of the pores first decreased and then increased with the increase in the w/c ratio. With the increase in the MSWI powder content, the average pore size of the foamed concrete first decreased and then increased, the average pore size and shape factor decreased, and the porosity changed a little, indicating that the MSWI powder had a limited role in regulating the pores, and the internal structure deteriorated when the content of MSWI powder was higher.
(4) When the dry density and w/c ratio changed, the pore structural parameters had a significant effect on the compressive strength, water absorption, and impermeability. The compressive strength of the foamed concrete decreased with the increase in the average pore size, the water absorption increased with the increase in porosity, and the impermeability decreased with the decrease in compressive strength.

## Figures and Tables

**Table 1:** The main chemical composition of the MSWI powder.

Composition	SiO_2_	CaO	Al_2_O_3_	Na_2_O	Fe_2_O_3_	P_2_O5	K_2_O	MgO	L.O.I
Content (%)	50.09	14.83	9.86	3.62	7.16	2.14	1.88	2.24	4.46

Note: L.O.I. indicates loss in ignition.

**Table 2:** Mixing proportions of the foamed concrete with MSWI powder.

Number	DD Designed (kg/m^3^)	Cement (kg)	MSWI (kg)	W/B Ratio	V_f_ (m^3^)	DD Tested (kg/m^3^)
G3F0W0.4	300	250	—	0.4	0.98	315 (±3)
G4F0W0.4	400	333	—	0.4	0.91	416 (±2)
G5F0W0.4	500	417	—	0.4	0.83	511 (±2)
G6F0W0.4	600	500	—	0.4	0.76	607 (±4)
G3F0W0.45	300	250	—	0.45	0.97	319 (±1)
G4F0W0.45	400	333	—	0.45	0.89	419 (±2)
G5F0W0.45	500	417	—	0.45	0.81	516 (±1)
G6F0W0.45	600	500	—	0.45	0.73	612 (±3)
G3F0W0.5	300	250	—	0.5	0.95	325 (±2)
G4F0W0.5	400	333	—	0.5	0.87	427 (±1)
G5F0W0.5	500	417	—	0.5	0.78	521 (±3)
G6F0W0.5	600	500	—	0.5	0.7	613 (±1)
G3F0W0.55	300	250	—	0.55	0.94	323 (±1)
G4F0W0.55	400	333	—	0.55	0.85	420 (±4)
G5F0W0.55	500	417	—	0.55	0.76	519 (±3)
G6F0W0.55	600	500	—	0.55	0.67	610 (±2)
G3F10W0.5	300	225	25	0.5	0.95	317 (±3)
G4F10W0.5	400	300	33	0.5	0.87	422 (±2)
G5F10W0.5	500	375	42	0.5	0.78	510 (±2)
G6F10W0.5	600	450	50	0.5	0.7	614 (±4)
G3F10W0.4	300	225	25	0.4	0.98	310 (±2)
G4F10W0.4	400	300	33	0.4	0.91	413 (±2)
G5F10W0.4	500	375	42	0.4	0.83	509 (±3)
G6F10W0.4	600	450	50	0.4	0.76	615 (±1)
G3F10W0.45	300	225	25	0.45	0.97	320 (±5)
G4F10W0.45	400	300	33	0.45	0.89	423 (±2)
G5F10W0.45	500	375	42	0.45	0.81	518 (±3)
G6F10W0.45	600	450	50	0.45	0.73	609 (±2)
G3F10W0.55	300	225	25	0.55	0.94	330 (±2)
G4F10W0.55	400	300	33	0.55	0.85	417 (±3)
G5F10W0.55	500	375	42	0.55	0.76	516 (±1)
G6F10W0.55	600	450	50	0.55	0.67	612 (±2)
G3F5W0.5	300	237.5	12.5	0.5	0.95	334 (±4)
G4F5W0.5	400	316	17	0.5	0.87	421 (±1)
G5F5W0.5	500	396	21	0.5	0.78	523 (±2)
G6F5W0.5	600	475	25	0.5	0.7	618 (±2)
G3F15W0.5	300	212.5	37.5	0.5	0.95	324 (±2)
G4F15W0.5	400	283	50	0.5	0.87	422 (±5)
G5F15W0.5	500	354	63	0.5	0.78	511 (±3)
G6F15W0.5	600	425	75	0.5	0.7	608 (±2)
G4F20W0.5	300	200	50	0.5	0.95	330 (±2)
G4F20W0.5	400	266	67	0.5	0.87	419 (±3)
G5F20W0.5	500	334	83	0.5	0.78	521 (±2)
G6F20W0.5	600	400	100	0.5	0.7	610 (±4)

Note: DD indicates dry density; V_f_ indicates the volume of the foams.

**Table 3:** Change rate of the compressive strength at different dry densities.

	MSWI	5%	10%	15%	20%
DD					
300 kg/m^3^		9.4%	22%	28.1%	40.4%
400 kg/m^3^		8.5%	21%	28.3%	32.4%
500 kg/m^3^		6.5%	18.9%	28%	31.7%
600 kg/m^3^		5.4%	17%	25.9%	29.9%

**Table 4:** Cumulative distribution of the apertures with different dry densities.

	Size	0.2 mm	0.4 mm	0.6 mm	0.8 mm	1.0 mm	>1.0 mm
DD (%)							
300 kg/m^3^		19	40	69	87	95	100
400 kg/m^3^		27.9	71.2	93.6	96	98.3	100
500 kg/m^3^		66.4	90.7	95.9	98.2	99	100
600 kg/m^3^		74.6	92.8	97.5	99	99.7	100

**Table 5:** Cumulative pore size distributions with different w/c ratios.

	Size	0.2 mm	0.4 mm	0.6 mm	0.8 mm	1.0 mm	>1.0 mm
W/B Ratio							
0.4		60.1	80.5	90.6	94.9	98	100
0.45		64.9	88.1	93.5	95.7	98.2	100
0.5		66.4	90.7	95.9	98.2	99	100
0.55		62.3	87.7	92.8	95.8	98.5	100

**Table 6:** Cumulative distribution of apertures with different dosages.

	Size	0.2 mm	0.4 mm	0.6 mm	0.8 mm	1.0 mm	>1.0 mm
MSWI							
0		61.3	88.5	94.9	97.1	98.5	100
5%		62.9	89.7	94.5	97.6	98.7	100
10%		66.4	90.7	95.9	98.2	99	100
15%		56.8	84.9	92.6	96.3	98.4	100
20%		53.7	83.2	91.5	96.1	98.1	100

## Data Availability

Data sharing is not applicable.
